# Association between D-dimer and in-hospital mortality risk in Acute Kidney Injury based on latent class dynamic trajectory

**DOI:** 10.3389/fmed.2025.1554213

**Published:** 2025-06-02

**Authors:** Lixiang Rao, Jiazheng Sun, Xingyang Zhao, Shuwang Ge, Ningxu Li

**Affiliations:** ^1^Division of Nephrology, Liyuan Hospital, Tongji Medical College, Huazhong University of Science and Technology, Wuhan, China; ^2^Division of Pneumology, Liyuan Hospital, Tongji Medical College, Huazhong University of Science and Technology, Wuhan, China; ^3^Division of Nephrology, Tongji Hospital, Tongji Medical College, Huazhong University of Science and Technology, Wuhan, China

**Keywords:** acute kidney injury, D-dimer, latent class trajectory model, in-hospital mortality risk, dynamic trajectories

## Abstract

**Objectives:**

To investigate the longitudinal D-dimer trajectories in hospitalized acute kidney injury (AKI) patients and analyze their association with in-hospital mortality risk.

**Methods:**

A retrospective study was conducted using data from AKI patients admitted to Tongji Hospital (July 2012–April 2024). General information, laboratory results, and outcomes were extracted from the medical record system. Patients with at least three D-dimer measurements within 30 days after AKI onset were included. Several latent class trajectory models (LCTMs) were constructed to identify distinct longitudinal dynamic trajectories of D-dimer. Model fit was assessed using Akaike Information Criterion, Bayesian information criterion, entropy, category probability and the optimal model was selected. Logistic regression and Kaplan-Meier survival analysis were employed to evaluate the relationship between D-dimer trajectories and in-hospital mortality. Subgroup analyses were performed to explore potential interactions between D-dimer trajectories and other variables.

**Results:**

Based on LCTMs evaluation, the model fitting indices were comprehensively analyzed, and a two-class model was identified as the optimal LCTM. The dynamic trajectories revealed two distinct patterns: an early peak followed by a gradual decline and a low-level continuous stability after AKI onset. Accordingly, patients were categorized into the high-peak decline group and the sustained low-level group. Logistic regression analysis demonstrated that AKI patients in the high-peak decline group had a significantly increased risk of in-hospital mortality (OR 2.27, 95% CI: 1.94–2.65). Kaplan-Meier survival curves indicated a reduced in-hospital survival rate in the high-peak decline group (*p* < 0.05). Subgroup analyses showed that, across age, gender, chronic kidney disease, cancer, surgery, myocardial infarction, and cerebral infarction subgroups, the high-peak decline group exhibited a significantly elevated risk of in-hospital mortality (*p* < 0.05), with no significant interaction effects observed among subgroups (*p* > 0.05).

**Conclusion:**

Using LCTM analysis, it was determined that D-dimer exhibits two characteristic longitudinal dynamic trajectories following AKI onset: an early peak followed by a gradual decline and a continuous low-level stability. Among these, the trajectory characterized by an early peak followed by a decline in AKI patients was associated with an increased risk of in-hospital mortality and reduced in-hospital survival, independent of age, gender, chronic kidney disease, cancer, surgery, myocardial infarction, or cerebral infarction.

## 1 Introduction

Acute Kidney Injury (AKI) is a prevalent critical illness associated with high mortality rates among hospitalized patients. The etiology of AKI is multifactorial, influenced by factors such as age, chronic kidney disease (CKD), pulmonary emboli, surgery and cancer. According to epidemiological data, approximately 13.3 million patients are diagnosed with AKI annually, resulting in 1.7 million deaths, with particularly high mortality observed in intensive care unit patients with AKI (1). Multinational and multicenter studies have demonstrated that the in-hospital mortality rate for AKI patients exceeds 60% (2). Moreover, the economic burden imposed by AKI-related mortality far surpasses that of other major diseases, including breast cancer, diabetes, and heart failure (3). Consequently, identifying a biomarker for assessing mortality risk in hospitalized AKI patients holds substantial clinical significance (4).

D-dimer, a soluble degradation product of cross-linked fibrin mediated by plasmin, is an emerging marker of AKI (5). Studies indicate that baseline D-dimer levels are closely associated with the risk of AKI-related mortality. Specifically, an increase in baseline D-dimer levels (D-dimer > 1,108 ng/mL) significantly elevates the risk of death in AKI patients, suggesting its potential as a predictive biomarker for AKI mortality risk (6). Importantly, as an economical, portable, and rapid biomarker, the longitudinal dynamic changes in D-dimer levels offer significant value in evaluating various diseases. For instance, cohort studies on cancer have shown that continuous monitoring of D-dimer dynamics before and after anticoagulant and thrombolytic therapy reveals that 70% of survivors exhibit a decrease in D-dimer levels. Conversely, patients without a reduction in D-dimer levels demonstrate a higher risk of mortality and systemic metastasis (7). However, current studies examining the relationship between D-dimer and AKI prognosis are limited to single-time-point measurements and fixed-threshold grouping, which may miss some key prognostic information.

Latent Class Trajectory Model (LCTM), an advanced statistical method derived from structural equation modeling, fits potential trajectory subgroups within observed data to intuitively describe the developmental trends of D-dimer and uncover its time-dependent patterns. By grouping patients based on distinct trajectories, LCTM addresses the limitations of traditional mean-based standard deviation approaches, which often neglect individual variability (8). This model has demonstrated significant value in various fields, including cognitive impairment (9), dietary intake (10), cancer (11), diabetes (12). Additionally, LCTM has been applied to AKI research, analyzing the relationship between serum creatinine change trajectories and AKI prognosis (13). In a prospective cohort study, the dynamic changes in renal tissue oxygen saturation were analyzed using a latent class model, revealing that patients with a gradual downward trend in renal tissue oxygen saturation exhibited a higher incidence of postoperative AKI, shorter survival, and increased mortality compared to those with continuously high levels (14).

Therefore, this retrospective cohort study introduces LCTM to analyze the dynamic change trajectory of D-dimer following AKI onset. By evaluating D-dimer levels at different time points, identifying potential change trends, and fitting distinct developmental trajectories, we aim to further assess the relationship between D-dimer dynamics and the risk of in-hospital mortality in AKI patients. This analysis provides a theoretical foundation for optimizing AKI treatment strategies.

## 2 Patients and methods

### 2.1 Patient characteristics

This is a retrospective cohort study. The clinical data of AKI patients admitted to Tongji Hospital Affiliated to Tongji Medical College of Huazhong University of Science and Technology from July 2012 to April 2024 were collected. Informed consent was exempted because of the retrospective nature of this study, and the study protocol was approved by the Ethics Committee of Tongji Hospital, Tongji Medical College, Huazhong University of Science and Technology (TJ-IRB202502003). According to the following criteria, AKI patients were screened and a total of 4,730 patients were included.

**FIGURE 1:**
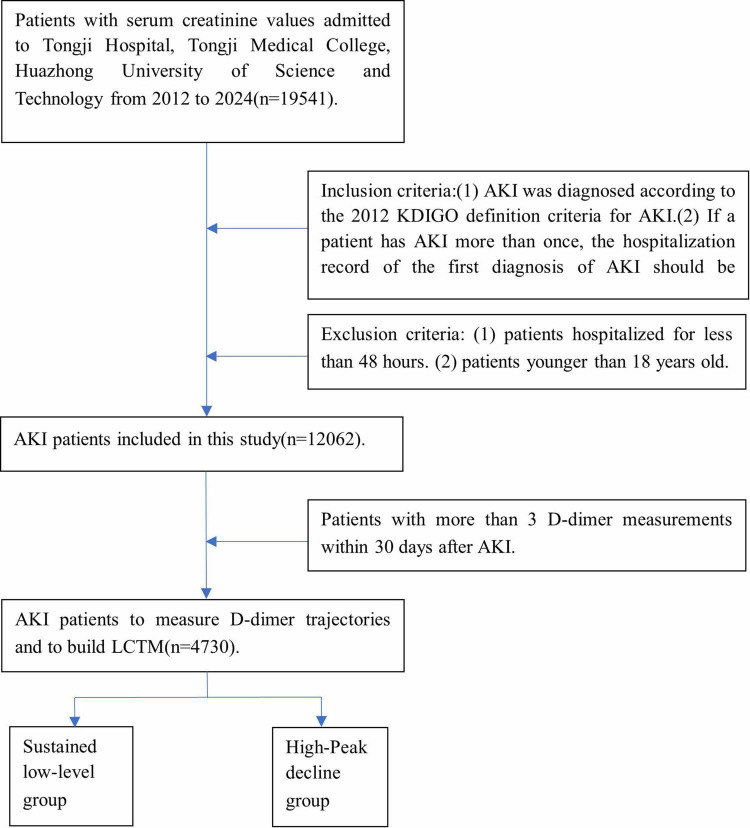
Flowchart of study patients.

Inclusion criteria: (1) AKI was diagnosed according to the 2012 KDIGO definition criteria for AKI (15): increased serum creatinine ≥ 0.3 mg/dL (≥ 26.5 μmol/L) within 48 h; An increase in serum creatinine to 1.5 or more times the baseline value, with known or presumed onset within the previous 7 days. (2) If a patient has AKI more than once, the hospitalization record of the first diagnosis of AKI should be retained. (3) Patients with more than 3 D-dimer measurements within 30 days after AKI. Exclusion criteria: patients hospitalized for less than 48 h. (4) Patients younger than 18 years old (Figure 1).

### 2.2 General information

The medical history data of the patients were collected through the electronic medical record data in the inpatient system. The general information included Length of hospital stay, Age, Sex, In-hospital mortality, comorbidities including Hypertension, Diabetes mellitus, Cancer, CKD, Myocardial Infarction, Cerebral Infarction, Blood disease, Pulmonary emboli, Surgery and Renal Replacement Therapy (RRT). Medication history included Heparin and Warfarin, Antiplatelet Agents, Thrombolytic Agents, Diuretics, and Chemotherapy drugs.

Laboratory parameters included White Blood Cell (WBC) count, Neutrophil count, Lymphocyte count, Hemoglobin, Platelet count, Alanine aminotransferase (ALT), Aspartate aminotransferase (AST), Albumin, Cholesterol, Total Bilirubin, Direct Bilirubin, Lactate dehydrogenase (LDH), Blood Urea Nitrogen (BUN), Serum Creatinine, Estimated Glomerular Filtration Rate (eGFR), Bicarbonate ion, and Calcium. Coagulation functions included Prothrombin Time (PT), Prothrombin Activity (PTA), International Normalized Ratio (INR), Activated Partial Thromboplastin Time (APTT), Thrombin Time (TT), Fibrinogen, and D-dimer.

D-dimer was detected by STAGO specific immunoturbidimetric assay, and sodium citrate was used for collection tube anticoagulation [Reference range: 0–0.5 μg/ml FEU (fibrinogen equivalent unit)].

### 2.3 Statistical analysis

For inpatients with AKI, D-dimer values were measured at least three times within 30 days following the onset of AKI for each patient. Given the varying time intervals between D-dimer measurements among patients, a standardized detection interval of 2 days was established. The mean value of all D-dimer measurements within each 2 days interval was calculated. Using the JMbayes package in R software (version 4.3.2), LCTMs with 1–5 classes were constructed, generating 1–5 subgroups representing distinct dynamic trajectory trends of D-dimer levels (referred to as D-dimer clusters) (Figure 1). These LCTMs were evaluated based on model fit using several indicators, including Log-likelihood value, Akaike Information Criterion (AIC), Bayesian Information Criterion (BIC), entropy, and Category probability. A higher log-likelihood value indicates better model-data fit, while lower AIC and BIC values suggest superior model performance. Entropy ranges from 0 to 1, with higher values reflecting better classification accuracy. Category probability represents the likelihood that an individual belongs to a specific D-dimer trajectory subgroup. Typically, the Category probability in each trajectory subgroup should not fall below 5%. Based on these evaluations, the optimal LCTM was selected, and a dynamic trajectory graph was generated. Patients were subsequently categorized according to their D-dimer trajectory patterns. Continuous variables were expressed as medians and quartiles and were analyzed by Kruskal-Wallis H test. Categorical variables were expressed as frequencies and percentages, and the chi-square test was used. Logistic regression analysis and Kaplan-Meier survival curves were employed to investigate the association between different D-dimer trajectory trends and in-hospital mortality risk among AKI patients. Subgroup analyses were conducted to explore the relationships between various covariates and D-dimer trajectory trends. All statistical tests were performed with the use of R software. *p* < 0.05 was considered statistically significant.

## 3 Results

### 3.1 Establishment and grouping of D-dimer longitudinal dynamic trajectory models

Sequentially, 1–5 models were established, generating 1–5 subgroups (clusters) representing the dynamic trajectory trends of D-dimer levels. As the number of clusters increased from 2 to 4, the Log-likelihood value progressively increased, while the AIC and BIC decreased. The highest entropy value reached 0.81, indicating a high degree of classification accuracy. However, when the number of clusters increased from 4 to 5, the increment in the Log-likelihood value and the decrement in AIC and BIC became marginal, accompanied by a decrease in entropy, suggesting that further increasing the number of clusters was not appropriate. Given that statistical indicators serve only as a reference for model selection, the interpretability of the model and the sample size distribution across clusters must also be considered. Notably, in the 3-cluster and 4-cluster models, the probability of certain classes fell below 5%, which may compromise the robustness of these models. After comprehensive calculation and analysis, the 2-cluster model was determined to be the optimal fit for LCTM (Table 1).

**TABLE 1:** Results of latent class trajectory model (LCTM) fit at the D-dimer level.

Model	Log-likelihood	AIC	BIC	Entropy	Category probability
1	−51433.09	102880.18	102925.41	–	1
2	−49191.69	98407.38	98484.92	0.78	74.61/25.39
3	−48279.68	96593.35	96703.20	0.81	69.60/3.28/27.13
4	−47891.84	95827.67	95969.83	0.81	67.95/25.43/2.49/4.12
5	−47658.09	95370.18	95544.65	0.78	65.43/12.80/15.05/2.37/4.36

AIC, Akaike Information Criterion; BIC, Bayesian Information Criterion.

**FIGURE 2:**
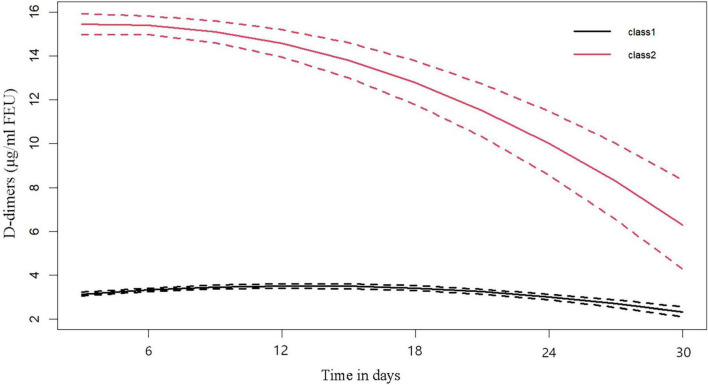
Dimer dynamic trajectories. “class 1” named as the “Sustained low-level group,” “class 2” named as the “high-peak decline group.”

In the optimal LCTM, the dynamic trajectory trend of D-dimer is categorized into two clusters, namely “Class 1” and “Class 2.” The figure indicates that in the Class 2 cluster, D-dimer levels peak during the early stages of AKI and subsequently exhibit a gradual downward trend, which is termed the “High-Peak decline Group.” In contrast, in the Class 1 cluster, D-dimer levels remain consistently low and stable, referred to as the “Sustained low-level Group” (Figure 2).

### 3.2 General characteristics

In-hospital mortality was significantly higher in the high-peak decline group compared to the sustained low-level group. Additionally, during the AKI stage, the proportion of AKI Stage 1 decreased, while the proportions of AKI Stage 2 and AKI Stage 3 increased in the high-peak Decline Group relative to the sustained low-level Group. Furthermore, the high-peak Decline Group exhibited higher proportions of pulmonary emboli, surgical interventions, CKD and RRT. Similarly, the usage rates of medications such as heparin and diuretics were also elevated in this group (Table 2).

**TABLE 2:** Baseline table of Acute Kidney Injury (AKI) in D-dimer dynamic trajectory clusters.

Variable	Sustained low-level group *n* = 3,529	High-Peak decline group *n* = 1,201	*P*
Age	59.00 (49.00, 68.00)	58.00 (49.00, 69.00)	0.827
**Sex**			
Male (%)	2,209 (62.60)	778 (64.78)	0.187
Female (%)	1,320 (37.40)	423 (35.22)	
Length of hospital stay (day)	18.00 (11.00, 29.00)	18.00 (10.00, 29.00)	0.103
In-hospital mortality (%)	511 (14.48)	333 (27.73)	< 0.001
**AKI stage (%)**			
1	2,160 (61.21)	511 (42.55)	< 0.001
2	886 (25.11)	362 (30.14)	
3	483 (13.69)	328 (27.31)	
**Comorbidities (%)**			
Hypertension	971 (27.51)	301 (25.06)	0.106
Diabetes mellitus	574 (16.27)	150 (12.49)	0.002
Cerebral infarction	394 (11.16)	148 (12.32)	0.300
Myocardial infarction	125 (3.54)	52 (4.33)	0.248
Blood diseases	413 (11.70)	70 (5.83)	< 0.001
Pulmonary emboli	56 (1.59)	37 (3.08)	0.002
Cancer	1,398 (39.61)	350 (29.14)	< 0.001
Surgery	658 (18.65)	333 (27.73)	< 0.001
CKD	1,635 (46.33)	628 (52.29)	< 0.001
RRT	657 (18.62)	429 (35.72)	< 0.001
**Medications (%)**			
Heparins	1,515 (42.93)	663 (55.20)	< 0.001
Warfarin	41 (1.16)	15 (1.25)	0.931
Thrombolytic agents	154 (4.36)	54 (4.50)	0.911
Antiplatelet agents	468 (13.26)	153 (12.74)	0.679
Diuretics	1,837 (52.05)	750 (62.45)	< 0.001
Chemotherapy agents	352 (9.97)	84 (6.99)	0.002
**Laboratory results**			
WBC count (10^9^/L)	7.17 (4.98, 11.00)	9.40 (6.22, 14.15)	< 0.001
Neutrophil count (10^9^/L)	6.47 (3.55, 10.92)	9.20 (6.11, 13.37)	< 0.001
Lymphocyte count (10^9^/L)	0.92 (0.53, 1.44)	0.73 (0.44, 1.15)	< 0.001
Platelet count (10^9^/L)	175.00 (104.00, 248.00)	127.00 (74.00, 196.00)	< 0.001
Hemoglobin (g/L)	108.00 (89.00, 128.00)	105.00 (88.00, 125.00)	0.007
ALT (IU/L)	20.00 (12.00, 40.00)	26.00 (15.00, 65.00)	< 0.001
AST (IU/L)	26.00 (17.00, 53.00)	47.00 (26.00, 117.00)	< 0.001
Albumin (g/L)	34.10 (29.40, 39.00)	33.00 (28.50, 37.00)	< 0.001
Cholesterol (mmol/L)	3.33 (2.47, 4.21)	3.00 (2.29, 3.89)	< 0.001
Bilirubin (μmol/L)	12.10 (7.40, 21.40)	16.20 (9.70, 32.10)	< 0.001
Direct bilirubin (μmol/L)	5.40 (3.30, 11.00)	7.70 (4.50, 17.90)	< 0.001
LDH (IU/L)	240.00 (178.00, 366.00)	399.00 (254.00, 674.00)	< 0.001
BUN (mmol/L)	6.40 (4.52, 9.60)	7.90 (5.30, 11.53)	< 0.001
Serum creatinine (μmol/L)	72.00 (54.00, 95.00)	76.00 (56.00, 102.00)	< 0.001
eGFR (mL/min/1.73 m^2^)	92.30 (67.40, 108.40)	87.90 (61.40, 106.50)	< 0.001
Bicarbonate ion	23.20 (20.60, 25.60)	22.20 (19.50, 24.80)	< 0.001
Calcium (mmol/L)	2.17 (2.03, 2.27)	2.12 (2.00, 2.24)	< 0.001
PT (second)	14.30 (13.10, 15.80)	15.30 (13.90, 17.50)	< 0.001
PTA (second)	81.00 (64.00, 96.00)	71.00 (54.00, 84.00)	< 0.001
INR	1.11 (1.00, 1.26)	1.21 (1.08, 1.44)	< 0.001
APTT (second)	39.40 (35.10, 45.80)	40.50 (35.70, 46.80)	0.003
TT (second)	16.60 (15.50, 17.90)	16.80 (15.40, 18.70)	0.001
Fibrinogen (g/L)	3.34 (2.43, 4.60)	3.04 (1.94, 4.38)	< 0.001

CKD, Chronic Kidney Disease; RRT, Renal Replacement Therapy; WBC, White Blood Cell; ALT, Alanine aminotransferase; AST, Aspartate aminotransferase; LDH, Lactate dehydrogenase; BUN, Blood Urea Nitrogen; eGFR, Estimated Glomerular Filtration Rate; PT, Prothrombin Time; PTA, Prothrombin Activity; INR, International Normalized Ratio; APTT, Activated Partial Thromboplastin Time; TT, Thrombin Time.

### 3.3 Association of D-dimer with the risk of in-hospital mortality in AKI

By logistic regression analysis, after adjusting for various covariates, we found that in assessing the in-hospital mortality risk of hospitalized AKI patients with different longitudinal dynamic trajectories of D-dimer, compared to the sustained low-level group, the in-hospital mortality risk was significantly higher in the high-peak decline group. Specifically, in the unadjusted model, the risk of in-hospital mortality in the high-peak decline group increased by 2.27 times (95% CI: 1.94–2.65). In Model 2, the important covariates were analyzed by referring to the bidirectional stepwise regression model. After adjustment, the in-hospital mortality risk in the high-peak decline group increased by 1.49 times (95% CI: 1.19–1.86). In Model 3, key covariates were determined based on LASSO regression. After adjustment, the in-hospital mortality risk for AKI patients in the high-peak decline group was 1.78 times (95% CI: 1.48–2.14) higher than that in the sustained low-level group (Table 3).

**TABLE 3:** Logistics regression analysis of D-dimer dynamic trajectory clusters on the risk of in- in-hospital mortality risk in patients with Acute Kidney Injury (AKI).

Group	Sustained low-level group	High-peak decline group	*P*
Model 1	Ref	2.27 (1.94–2.65)	< 0.001
Model 2	Ref	1.49 (1.19–1.86)	< 0.001
Model 3	Ref	1.78 (1.48–2.14)	< 0.001

Model 1 did not adjust the model. Model 2, through a two-way stepwise regression model, covariates were adjusted such as Age, Aki Stage, Cancer, Diabetes, Surgery, RRT, Warfarin, Diuretics, Chemotherapy Drugs, Neutrophils count, Lymphocytes count, Platelet count, ALT, Albumin, LDH, BUN, eGFR, Bicarbonate ion and PTA. Model 3 through the LASSO model, adjust co-variables such as Age, Myocardial Infarction, Blood Disorders, Hypertension, Surgery, RRT, Diuretics, WBC count, Neutrophils count, Lymphocytes count, Hemoglobin, Platelet count, ALT, AST, LDH, Albumin, Total Bilirubin, Direct Bilirubin, Cholesterol, Creatinine, eGFR, BUN, Bicarbonate ion, Calcium, and PT, TT, INR, PTA, APTT.

### 3.4 Kaplan-Meier survival curve

**FIGURE 3:**
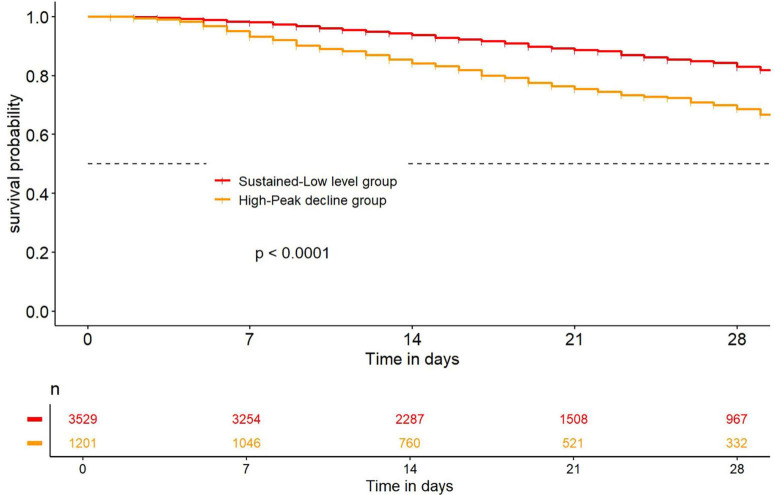
Kaplan-Meier survival curve with D-dimer dynamic trajectory clusters.

After LCTM analysis, the Kaplan-Meier survival curve showed that the in-hospital survival rate of hospitalized AKI patients in the high-peak decline group was significantly lower than that in the sustained low-level group (*p* < 0.05), indicating that the in-hospital survival rate was lower when the initial value of D-dimer was high after AKI (Figure 3).

### 3.5 Subgroup analysis

The high-peak decline group exhibited a significantly higher risk of in-hospital mortality compared to the sustained low-level group, irrespective of whether AKI patients were older than 60 years, male or female, or had comorbidities such as CKD, cancer, surgery, myocardial infarction, or cerebral infarction. No significant interaction was observed among the subgroups (*p* > 0.05). Specifically, the risk of mortality for patients with AKI combined with myocardial infarction increased by 3.10 times (95% CI: 1.49–6.42), while the risk of in-hospital mortality for patients with AKI combined with cancer increased by 3.09 times (95% CI: 1.85–5.17). Additionally, no significant correlation was found between the in-hospital mortality risk in the high-peak decline group and the sustained low-level group among AKI patients with pulmonary emboli (OR 1.93, 95% CI: 0.67–5.57) (Table 4).

**TABLE 4:** Subgroup analysis of the D-dimer dynamic trajectory clusters on the risk of in-hospital mortality risk in patients with Acute Kidney Injury (AKI).

Variable		*N* (%)	Sustained low- level group	High-peak decline group	*P*	*P* interaction
Age						0.642
	≤ 60	2,484 (53%)	Ref	2.51 (1.92–3.28)	< 0.001	–
	> 60	2,246 (47%)	Ref	2.72 (2.19–3.39)	< 0.001	–
Sex						0.938
	Male	2,987 (63%)	Ref	2.54 (2.08–3.12)	< 0.001	–
	Female	1,743 (37%)	Ref	2.51 (1.88–3.35)	< 0.001	–
CKD						0.239
	No	2,467 (52%)	Ref	2.79 (2.17–3.58)	< 0.001	–
	Yes	2,263 (48%)	Ref	2.28 (1.82–2.85)	< 0.001	–
Cancer						0.089
	No	2,982 (63%)	Ref	2.26 (1.85–2.76)	< 0.001	–
	Yes	1,748 (37%)	Ref	3.09 (2.29–4.18)	< 0.001	–
Surgery						0.460
	No	3,739 (79%)	Ref	2.80 (2.33–3.37)	< 0.001	–
	Yes	991 (21%)	Ref	2.35 (1.53–3.61)	< 0.001	–
Pulmonary emboli						0.608
	No	4,637 (98%)	Ref	2.55 (2.16–3.02)	< 0.001	–
	Yes	93 (2%)	Ref	1.93 (0.67–5.57)	0.225	–
Myocardial infarction						0.580
	No	4,553 (96%)	Ref	2.50 (2.11–2.97)	< 0.001	–
	Yes	177 (4%)	Ref	3.10 (1.49–6.42)	0.002	–
Cerebral infarction						0.090
	No	4,188 (88%)	Ref	2.68 (2.24–3.21)	< 0.001	–
	Yes	542 (12%)	Ref	1.75 (1.11–2.77)	0.017	–

CKD, Chronic Kidney Disease.

## 4 Discussion

In this study, 1–5 LCTMs were established. Based on model evaluation indicators such as BIC and AIC, the LCTM in cluster 2 was ultimately selected as the optimal model. The dynamic trajectory diagram revealed that D-dimer levels in the best LCTM exhibited two characteristic evolution trends following AKI onset: the high-peak decline group, characterized by an early peak value followed by a time-dependent gradual decrease, and the sustained low-level group, which maintained a stable state of low values. Logistic regression analysis demonstrated that in-hospital mortality risk in the high-peak decline group was significantly increased (OR 2.27, 95% CI: 1.94–2.65). Kaplan-Meier survival curves further indicated that the short-term hospital survival rate of patients in the high-peak decline group was significantly lower than that in the control group (*p* < 0.05). Subgroup analysis revealed no significant interaction among subgroups based on age, sex, CKD, cancer, surgery, myocardial infarction, or cerebral infarction (*p* > 0.05).

Some studies have identified D-dimer as an emerging marker for AKI (5), with its baseline level holding important clinical significance in the occurrence and progression of AKI. Higher baseline D-dimer levels are associated with an increased risk of AKI (16). When the baseline D-dimer level exceeds 2.2 mg/L, it becomes a risk factor for AKI (17). Elevated baseline D-dimer levels can also predict AKI progression, with an Area Under Curve (AUC) of 0.755 (95% CI: 0.718–0.793) (18). Furthermore, D-dimer is strongly associated with mortality risk in AKI patients. Studies have shown that an increase in baseline D-dimer levels (D-dimer > 1,108 ng/mL), particularly when combined with a hypercoagulable state, significantly increases the risk of death in pregnant patients with AKI, with an AUC of 0.828 (95%CI: 0.670–0.986) (6). However, these studies are limited to baseline assessments and suffer from the disadvantage of single-time-point detection or fixed-threshold grouping (19), which fails to reflect the dynamic imbalance between the coagulation system and secondary fibrinolytic system.

By introducing LCTM, this study identified dynamic track changes associated with disease prognosis. Previous studies found that D-dimer exhibited two trends before and after thrombolytic therapy in patients, where patients whose plasma D-dimer levels rapidly increased, peaked, and then rapidly declined to preoperative levels achieved complete recanalization of lower limb arteries (20). Additionally, some studies have shown that COVID-19 patients with stable D-dimer trajectories had hazard ratios (HRs) of 0.29 (95% CI: 0.17–0.49, *p* < 0.0001) relative to those with increasing D-dimer trajectories for mortality outcomes (21). This study revealed two potential trends in D-dimer dynamics following AKI onset: one where D-dimer peaks early and gradually decreases over time, classified as the high-peak decline group, and another where D-dimer remains consistently low within 30 days post-AKI, classified as the sustained low-level group. Patients with an initial peak D-dimer value exhibit a lower in-hospital survival rate and higher in-hospital mortality risk, suggesting that the early peak in D-dimer during AKI may result from the interaction between enhanced early coagulation function and hyperfibrinolysis or partial dissolution of microthrombi. The subsequent decline could be attributed to therapeutic intervention or compensatory hyperfibrinolysis. Conversely, persistent low-level status indicates minimal coagulation activation and less severe tubular injury. Importantly, this finding remained unaffected by age, sex, CKD, cancer, surgery, myocardial infarction, or cerebral infarction, underscoring the broad universality of the prognostic value of D-dimer dynamic trajectories.

This mechanism aligns with the pathological process of coagulation dysfunction observed in liver cirrhosis with renal injury (22). From a pathophysiological perspective, when the imbalance between coagulation and anticoagulation systems leads to hypercoagulation, the probability of thromboembolism in the renal microcirculation system significantly increases (23). Studies have shown that the hypercoagulable state can cause microvascular fibrin deposition (24), which in turn increases renal microthrombosis (23). The early peak in D-dimer may indicate the formation of renal microthrombi, followed by compensatory activation of the endogenous fibrinolytic system (25). Impaired clearance of microthrombi results in glomerular capillary network ischemia-reperfusion injury. Oxidative stress and inflammatory mediators such as Interleukin- 6 (IL-6) and Tumor Necrosis Factor-α (TNF-α) subsequently promote renal tubular cell apoptosis (26), leading to decreased glomerular filtration rate and renal tubular reabsorption disorder, thereby exacerbating kidney injury (27).

This study has limitations. This is a single-center retrospective cohort study. First, Single-center studies are limited by the geographical location and population distribution of the specific institution. Caution should be exercised when extrapolating the research conclusions to other regions. Second, the retrospective study inherently limits causal inference. Additionally, this retrospective study lacks data on inflammatory markers such as IL-6, C-reactive protein (CRP), and Procalcitonin (PCT), as well as standardized clinical severity scores like SOFA and APACHE II. The absence of these critical variables may have obscured the independent contributions of multi-organ dysfunction and systemic inflammatory responses to in-hospital mortality risk among AKI patients, potentially leading to an overestimation of the predictive value of D-dimer dynamics for mortality risk. Furthermore, AKI diagnosis was based solely on serum creatinine levels without incorporating urine output criteria, which could result in underdiagnosis of early or mild AKI cases. And the lack of follow-up data after discharge also lacks a comprehensive long-term prognosis analysis. Additionally, the study found no significant association between pulmonary emboli and the longitudinal dynamic changes in D-dimer levels. The limited sample size of pulmonary emboli cases in the subgroup analysis may have compromised statistical power, highlighting the need for further investigation with a larger cohort. Future studies should consider adopting a multi-center prospective design, standardizing measurement protocols, systematically collecting urine output data, and integrating multiple dynamic indicators such as coagulation factors and inflammatory markers to strengthen the robustness and clinical relevance of the evidence.

In conclusion, longitudinal dynamic trajectory analysis of D-dimer revealed two distinct trajectories following AKI onset. Compared with the continuously low-level stable trajectory, patients in the early peak value and gradually decreasing trajectory exhibited a lower in-hospital survival rate and a significantly increased risk of in-hospital mortality. These findings were unaffected by age, sex, CKD, cancers, surgery, myocardial infarction, or cerebral infarction. D-dimer offers the advantages of economic and rapid, making it a valuable reference for prognosis evaluation and individualized treatment decisions in AKI patients.

## Data Availability

The original contributions presented in the study are included in the article material, further inquiries can be directed to the corresponding authors.
